# The effect of service-based learning on health education competencies of students in community health nursing internships

**DOI:** 10.1186/s12912-024-01799-y

**Published:** 2024-02-23

**Authors:** Maryamsadat Emrani, Zohreh Khoshnood, Jamileh Farokhzadian, Mohammad Sadeghi

**Affiliations:** 1 Student Research Committee, Razi faculty of Nursing and Midwifery, Kerman University of Medical Sciences, Kerman, Iran; 2 Department of Community Health Nursing, Razi Faculty of Nursing and Midwifery, Kerman University of Medical Sciences, Kerman, Iran; 3 Nursing Research Center, Kerman University of Medical Sciences, Kerman, Iran; 4 Education development center, Sirjan school of medical sciences, Sirjan, Iran

**Keywords:** Service-based learning, Health education competencies, Students, Nursing education, Nursing internships

## Abstract

**Aim:**

This study investigated the impact of service-based learning on the health education competencies of students in community health nursing internships. community health nursing internship is one of the areas where students acquire health education competencies. Studies have shown that some students have poor health education competencies, and new educational interventions, such as service-based learning, can help improve their competencies.

**Method:**

This quasi-experimental study was conducted in 2021–2022. The participants were final-year nursing students affiliated to Kerman University of Medical Sciences. All participants (*n* = 72) were selected via the census method and randomly divided into intervention and control groups (36 participants in each group). The students in the intervention group attended a service-based learning program for 20 days. The data were collected before and one month after intervention using a 48-item health education qualification questionnaire. The collected data were analyzed using SPSS22 software.

**Results:**

The results showed that the mean health education competencies scores were lower before intervention in the intervention and control group (165.75 ± 23.09) (170.16 ± 28.58)(*p* > 0.05). There was no significant difference between the two groups in terms of their mean scores on health education competencies(*p* > 0.05). The health education competencies score increased significantly for the participants in the intervention group (191.58 ± 28.35) compared to the control group (165.97 ± 28.11) after intervention.

**Conclusion:**

Nursing administrators and professors need to take effective steps to empower nursing students as much as possible and incorporate service-based learning techniques in clinical education programs for nursing students.

## Introduction

Nursing is a unique profession because nurses assume many simultaneous roles, such as providing direct care, making clinical decisions, supporting clients and families, teaching, and acquiring necessary skills in clinical education [1]. Clinical education is a distinctive process because it allows for practical application of knowledge in real-life situations. Additionally, acquiring professional competencies, particularly health education competencies, is not possible through theoretical courses alone [2]. Health education competencies refer to a set of knowledge, attitudes, and skills required for success in health education and encompass competencies such as clinical, educational, technical, and social skills [3]. Apart from practical skills and knowledge, nurses should possess effective problem-solving, decision-making, and communication skills, as well as the ability to make sound judgments in different situations [4].

Currently, new teaching methods receive much attention in theoretical and clinical education programs, as traditional teaching methods alone are less effective in training students to acquire necessary competencies. One of these modern methods is service-based learning. Service-based or outcome-based learning has gained importance in professional health curricula, including nursing, aiming to educate students about social responsibility and train individuals who can respond to the community’s expectations [5]. Service-based learning is both an educational program and philosophy. In the educational program, learners provide meaningful services to their community while involving community members, and in the educational philosophy, they learn about social responsibility [6]. This type of learning involves students in experiential activities that contribute to their growth, expose them to clients from different cultures, and enable them to provide effective services for people in diverse cultures [7]. The goals of service-based learning include promoting learning through university educational programs and instilling moral values and professional commitments in students as they engage in providing services and meeting real community needs [8].

The essential elements of service-based learning include obligation, critical thinking, being two-sided, leadership development, and meaningful service experience. In service-based learning programs, students commit to carrying out planned service, critically analyze it, and identify real public problems to develop citizenship commitment skills, effective planning, and appropriate leadership skills [9]. The seven steps of implementing service-based learning include meeting the community’s known needs, achieving curriculum goals through a new service-oriented learning approach, fully reflecting the service-oriented learning experience, developing and promoting students’ responsibility, establishing and enhancing community participation, planning, and finally equipping students with knowledge and skills [10]. The components of service-based learning include establishing a partnership between the community and the faculty, expressing and clarifying the outcomes and competencies obtained from the service-based learning process, selecting textbooks and other learning resources, providing educational programs, designing evaluations, designing infrastructures, and maintaining and updating course lessons and required activities [11].

If service-based learning is provided effectively, it offers several advantages and benefits to learners, the community, and educational institutions. Learners address the real needs of the community and gain positive, meaningful, and authentic experiences. Moreover, they develop skills in group work, responsibility, mutual dependence, altruism, emotional outcomes, a sense of usefulness and efficiency, as well as cognitive, emotional, and social learning. Service-based learning empowers learners to face social problems and handle complex situations. Thus, the benefits of service-based learning can be categorized into three main categories: personal development, social responsibility, and educational benefits, including improved problem-solving skills, enhanced speaking and reading abilities, higher academic achievement, and increased concentration and sense of academic achievement. Community-related benefits include attention to and addressing community needs, additional human resources, active citizenship, empowerment, and improved community properties. Educational institutions benefit from service-based learning by training motivated learners, fostering a cooperative learning environment, and producing employees with rational thinking [12].

A review of the literature suggests that many students lack theoretical knowledge, practical skills, and effective communication abilities before entering the clinical setting or are not familiar with the clinical setting before starting the internship program [13].

Another study reported that most teachers and students had a relatively poor assessment of internship programs in terms of helping students acquire a social perspective and gain nursing skills for patient care. Additionally, students and teachers faced problems such as limited access to welfare and educational facilities, non-cooperation of healthcare teams, dispersion of internships across clinical departments during the program, and they believed that changes should be made to the organization and execution of internship courses [14].

Dadgaran et al. (2013) highlighted the need for early prevention and simultaneous evaluation of the service-based learning method in nursing students and showed that this method helped people gain a deep understanding of differences and strengthen their knowledge, skills, and communication abilities [15]. Khorrami Rad et al. (2011) examined the impact of the service-based learning method and found that it increases students’ awareness and changes their attitudes toward usual methods [16]. Hwang et al. (2020) also stated that service-based learning significantly increased the critical thinking ability of all students [17]. Dombrowsky et al. (2019) found that Service-learning fosters student creativity and independence and is more focused on client or agency need. Also provides a broader perspective of health care and an increased sense of agency and self-confidence [18]. Furthermore, Read et al. (2018) showed that implementing service-based learning in schools using recommended strategies and curriculum content leads to coherent, integrated learning and a successful experience for students [19]. Hwang et al. (2014) also showed that service-based learning, when implemented with sufficient training, facilitates students’ learning [20].

In general, previous studies have shown that service-based learning is an integral element of nursing education. Despite the advantages of service-based learning, the studies have highlighted some obstacles and challenges faced by students, medical colleges, and schools, including limited opportunities and time, the unpredictability of the real world, mismatch with students’ learning styles, heavy workload, limited infrastructure and support, lack of financial and human resources, and coordination and planning problems. Specific challenges faced by students include procrastination, fear of unpreparedness, severe anxiety in dealing with challenging clients, and feelings of depression and despair in learning environments. These challenges require further studies on the effectiveness of service-based learning [21, 22].

Thus, based on the literature, one of the goals of health internship programs is to train community-oriented nurses and provide effective nursing services to community members. Additionally, there is a need for training creative and skilled students, improving their competencies in health internships, and enhancing their knowledge, attitudes, and social skills in the nursing profession. To this end, the present study aimed to investigate the effect of service-based learning on the health education competencies of students in community health nursing internships.

## Method

This quasi-experimental study was conducted during the first and second semesters of the academic year 2021–2022. The participants were selected from last-year nursing students at Razi School of Nursing and Midwifery, which is affiliated with Kerman University of Medical Sciences in southeast Iran. The students were selected using the census method and were then randomly divided into intervention and control groups, with 36 students in each group. The research setting consisted of two health centers where the students completed their community health nursing internships.

Initially, all last-year nursing students (*N* = 72) were selected using the census method. Then, they were randomly divided into two groups, control and intervention, by drawing lots (36 students in each group). After explaining the study process to students, researcher obtained their informed consent. The criteria for enrollment in the study were willingness to participate, completion of a nursing undergraduate program, and successful completion of the health internship in healthcare centers. The exclusion criteria included unwillingness to continue participating in the study, lack of active participation in the internship course, and being an exchange student.

As part of the academic program, the students completed their internships every month in groups of 4 people at two comprehensive health centers. Each theoretical training session lasted for 45 min. The students in the intervention group then participated in the community nursing internship program for a total of 20 days spread across eight sessions. During their time at the health centers, the students first identified community and public needs in a specific area through discussions and exchange of opinions. Subsequently, they developed the necessary planning after setting the goals. The students also received theoretical training on community problems. Following this, the students attempted to solve the identified problems and provide services while engaging with the respective area. Finally, the services provided by the students were assessed.

On the other hand, the students in the control group received usual training, while the participants in the intervention group attended the service-based learning program and put the instructions into practice within the community. In the second semester, the control group enrolled in the study with the same number and grouping as the intervention group. The difference between the control and intervention groups was that the students in the control group were taught only theoretically and were not looking for a solution to meet the needs of the society. the intervention program was adapted based on the number of internship days. A summary of the instructions and content of the intervention program was presented in the last session. Table 1 provides an overview of the instructions provided in the service-based program.

**Table 1:** A summary of the instructions provided in the intervention program

Sessions	Instructional content
1	Introducing the health system Introducing the integrated health system (SIB)
2	Happy children program (healthy child, monitoring the child’s growth and nutrition, and promoting breastfeeding)
3	Supplemental assistance to 2-5-year-old children Varnish (fluoride therapy) for children aged 3 to 13 years Risk assessment in terms of blood pressure, blood sugar, blood lipids, and BMI Screening cervix, breasts, intestine, AIDS, hepatitis, tuberculosis, cholera, and lice Universal neonatal screening (G6PD enzyme deficiency, phenylketonuria, hypothyroidism, and hearing)
4	Prevention of disasters and accidents
5	Vaccination, cold chain, child development, and health
6	Maternal healthcare program Adolescent, youth, and student health program Middle-aged health program Elderly health program
7	Infectious and non-infectious disease control program
8	Introducing the activities of the nutrition and service delivery unit Introducing the activities of the mental health and service delivery unit Introducing the activities of the oral health and service delivery unit Introducing the activities of the environmental health and service delivery unit

The data in this study were collected using a demographic information questionnaire and the health education competencies questionnaire. The demographic information questionnaire contained items that assessed the participants’ personal and occupational characteristics, such as age, gender, marital status, place of residence, province of residence, completion of service-based learning courses in the past, its impact on their future career, and the extent to which they were interested in nursing work.

The health education competencies questionnaire contained 48 items and 5 subscales: skills (10 items), knowledge (19 items), community presence (6 items), attitudes (6 items), and professional preparation (6 items). A respondent’s score ranged from 48 to 240. The items were scored using a 5-point Likert scale ranging from very poor to very good (1 = very poor, 2 = poor, 3 = moderate, 4 = good, and 5 = very good). The content validity of the Persian version of the questionnaire was assessed by 10 professors at the nursing school of Kerman University of Medical Sciences. They rated the items in terms of simplicity, clarity (qualitatively), and relevance (quantitatively). The content validity index (CVI) was equal to 0.84, and the reliability of the tool was estimated using the internal correlation method and Cronbach’s alpha coefficient (α = 0.75) by administering the questionnaire to 30 participants before the intervention. The results confirmed the reliability and validity of the instrument [3].

The validity of this questionnaire was assessed by Creswell (2007). The instrument was the Arabic and English version of the questionnaire, which was assessed by a group of 10 managers and senior Saudi nursing experts at the University of Salford and academic staff at the University of Dammam. The reliability of the instrument was assessed using Cronbach’s alpha and a 50-item Likert scale. The value of Cronbach’s alpha for each component was higher than 0.70, confirming the internal consistency of the instrument [3].

The data were collected from the students in the intervention group before and one month after the intervention, and from the participants in the control group before and one month after completing the internship program. The collected data were analyzed using SPSS22 software, employing the chi-square test, paired samples t-test, and independent samples t-test. The Kolmogorov-Smirnov test was used to check the normality of the data.

One of the limitations of this study was students unwilling to participate, so the researcher considered one session off from the internship for the students who participated in the study. Another limitation was the information exchange between the control and intervention groups, so the students in the intervention group asked to prevent the spread of information to the control group.

## Results

The participants were 72 nursing students, divided into two groups: the intervention group and the control group, each consisting of 36 students. The data from the chi-square test and independent samples t-test indicated that the students in both the intervention and control groups were homogeneous in terms of the demographic variables and showed no significant differences (Table 2).

**Table 2:** A comparison of the demographic variables between the two groups

Variable	Grouping	Intervention group		Control group		Statistic	*P*-value
		n	%	n	%		
Gender	Male	18	50%	16	44.4%	χ^2^ = 0.22	0.63
	Female	18	50%	20	55.6%		
Marital status	Single	29	80.6%	31	86.1%	χ^2^ = 0.4	0.52
	Married	7	19.4%	5	13.9%		
Place of residence	Personal house	15	41.7%	22	61.1%	χ^2^ = 2.72	0.09
	Dormitory	21	58.3%	14	38.9%		
Residing in Kerman province	Yes	22	61.1%	22	61.1%	χ^2^ = 0.00	1
	No	14	38.9%	14	38.9%		
Completing a service-based learning course in the past	No	22	61.1%	25	69.4%	χ^2^ = 0.72	0.77
	Nursing education	10	27.8%	8	22.2%		
	Training sessions	2	5.6%	1	2.8%		
	Non-nursing education	1	2.8%	0	0		
	Attending conferences and seminars	1	2.8%	2	5.6%		
Interested in nursing	Greatly	16	44.4%	9	25%	χ^2^ = 012	0.72
	Moderately	16	44.4%	21	58.3%		
	Little	4	11.1%	5	13.9%		
	Very little	0	0	1	2.8%		
The effectiveness of service-based learning in future career	Yes	32	88.9%	31	86.1%	χ^2^ = 2.91	0.40
	No	4	11.1%	5	13.9%		
Age		23.13 ± 2.46		22.11 ± 0.94		t = -0.15	0.88

The mean health education competencies score for the students in the intervention group before the intervention (165.7 ± 23.09) was lower than mean score of the students in the control group in the post-test (170.16 ± 28.58). However, the data from the independent samples t-test indicated no significant difference between the two groups in the mean scores of health education competencies and the scores on the related subscales.

In contrast, the mean health education competencies score for the students in the intervention group after the intervention (191.58 ± 28.35) was higher than mean score of the students in the control group in the post-test (165.97 ± 28.11). Additionally, the independent samples t-test showed significant differences between the two groups in the mean scores of health education competencies and the scores on the related subscales (Table 3).Analysis of covariance was performed to control for the effects of the pre-test on the post-test scores of health education competencies. The results indicated that the significant increase in the health education competencies of the intervention group was attributed to the service-based learning method. These findings corroborate the results presented in Table 3 (Table 4).

**Table 3:** A comparison of the scores of health education competencies and their subscales between the two groups

Variable	Groups	Pre-intervention	Post-intervention	Mean difference	Effect size	Paired t-test	*p*- value
Health education competencies	Intervention	165.75 ± 33.09	191.58 ± 28.35	25.83	0.83	-4.96	**0.001**
	Control	170.28.58 ± 33.09	165.97 ± 28.11	4.19	0.14	0.76	0.44
	Independent t-test *p*- value	-0.60 0.54	3.84 0.001				
	Effect size	0.14	0.90				
Skills	Intervention	35.61 ± 7.01	40.94 ± 6.25	5.33	0.80	-4.37	**0.001**
	Control	35.25 ± 6.04	34.91 ± 5.41	0.33	0.05	0.38	0.7
	Independent t-test *p*- value	0.23 0.81	4.37 0.001				
	Effect size	0.05	1.03				
Knowledge	Intervention	63.91 ± 14.41	75.75 ± 12.53	11.3	0.87	-5.32	**0.001**
	Control	66.69 ± 12.32	64.88 ± 12.08	1.8	0.14	0.70 0.48	
	Independent t-test *p*- value	-0.87 0.38	3.74 0.001				
	Effect size	0.20	0.88				
Community presence	Intervention	19 ± 5.16	22.13 ± 3.85	3.13	0.68	-3.97	**0.001**
	Control	20.02 ± 4.72	20.25 ± 4.30	0.22	0.05	-0.24	0.18
	Independent t-test *p*- value	-0.88 0.38	1.98 0.05				
	Effect size	0.20	0.46				
Attitudes	Intervention	20.61 ± 4.34	23.611 ± 3.78	3	1.72	-4.28	**0.001**
	Control	21.55 ± 4.09	20.47 ± 4.07	1.08	0.26	1.31	0.19
	Independent t-test *p*- value	-0.94 0.34	3.38 0.01				
	Effect size	0.22	0.79				
Professional preparation	Intervention	26.61 ± 4.98	29.13 ± 4.21	4.21	2.52	-3.04	**0.004**
	Control	26.63 ± 4.72	25.44 ± 4.88	1.19	0.24	1.43	0.16
	Independent t-test *p*- value	-0.02 0.98	3.43 0.001				
	Effect size	0.004	0.80				

**Table 4:** The results of analysis of covariance for two intervention and control groups

	Type III sum of squares	df	Mean square	F	*p*-value
Intercept	1182.73	2	1182.73	44.55	**0.001**
Skills	561.33	1	561.33	21.15	**0.001**
Group	620.07	1	620.07	23.36	**0.001**
Error	1831.30	69	26.54		
Intercept	6400.14	2	6400.14	13.9	**0.001**
Knowledge	1534.24	1	1534.24	48.64	**0.001**
Group	2492	1	2492	11.66	**0.01**
Error	907.08	62	131.56	18.94	
Intercept	1004.46	2	1004.46	68.11	**0.001**
Community presence	151.51	1	151.51	10.27	**0.002**
Group	85.72	1	85.72	5.81	**0.02**
Error	1017.54	62	14.74		
Intercept	594.83	2	594.83	43.85	**0.001**
Attitudes	145.55	1	145.55	10.73	**0.002**
Group	212.93	1	212.93	15.69	**0.001**
Error	935.97	62	13.56		
Intercept	595.57	2	595.57	34.96	**0.001**
Professional preparation	284	1	284	16.67	**0.001**
Group	247.21	1	247.21	14.51	**0.001**
Error	1175.19	62	17.03		
Intercept	30373.45	2	30373.45	45.4	**0.001**
The total of competence of health education	9645.77	1	9645.77	14.41	**0.001**
Group	13333.46	1	13333.46	19.93	**0.001**
Error	46157.97	62	668.95		

## Discussion

This study examined the effect of service-based learning on the health education competencies of students in the community health nursing internship program. The results showed that service-based learning positively affects the health education competencies of the nursing students in the intervention group. Similarly, Dadgaran et al. (2013) stated that the service- based learning method helps students work in different settings such as social health institutions. Thus, they deal with special populations such as older adults. The students can also develop a deep understanding of differences and cultural issues among older adults and promote their knowledge, abilities, and communication skills [15].

Avazeh et al. (2014) also found out service-based learning will increase nurses’ efficiency and quality of care [23]. Khorrami Rad et al. (2011) reported that the scores of knowledges and attitudes of the students in the intervention group after using the service-based learning method were significantly higher than the mean scores for the same variables in the control group. Moreover, the mean scores of knowledges for the students in the intervention group showed a significant difference before and after the intervention [16]. Valizadeh et al. (2010) showed that outcome-based education is effective in improving students’ cognitive and behavioral skills [24]. Hwang et al. (2020) also reported that the global health competencies, self-assessed global leadership, and critical thinking ability of all students increased significantly after training. Thus, nursing educators and global health experts can use discussion and exchange of opinions for educating students and solving their problems [17]. Rosen et al. (2019) also confirmed the positive effects of service-based learning such as interdisciplinary training and effective communication [25]. Dombrowsky et al. (2019) also suggested that the development of skills, teamwork, and leadership and the application of theory in practice with the service-based learning method increased the creativity and independence of health students and made them focus on clients and understand them better [18]. Read et al. (2018) found that implementing service-based learning in schools using the recommended strategies along with the curriculum content of the students will lead to coherent, integrated learning and a successful experience for them [19]. Hayward et al. (2017) showed that planning, implementation, and evaluation are highly efficient to maximize the benefits of service-based learning [26].

Knecht et al. (2015) also emphasized that listening to the voices of students who attend service-based learning courses enables teachers to gain a deeper perspective of their experiences and ultimately leads to a deeper understanding of service-based learning programs [27]. Hwang et al. (2014) showed that the perceived care was significantly different between the students and paired residents in the intervention group and the control group and the knowledge and attitude scores of nursing students increased significantly. The results of this research project confirmed that students can learn more effectively with adequate training and the effective use of service-based learning [20]. Furthermore, Long et al. (2014) showed that service-based learning effectively increased the self-efficacy, self-confidence, skills, knowledge, attitude, and self-awareness of students in working with Spanish culture and developed their cultural competence [28].

## Conclusion

The present study confirmed the effectiveness of service-based learning on the health education competencies of students in the community health nursing internship program. Thus, following the results of the present study and similar studies in the literature, the outcome-based learning method improves nursing students’ competencies compared to traditional methods. Furthermore, since nursing students are interested in participating in service-oriented activities, it is necessary to provide them with the opportunity to learn theoretical and practical concepts together and to shift learning from passive and traditional learning to active and modern learning. In other words, the effective application of service-based learning in nursing universities can contribute to recognizing students’ educational problems. Furthermore, a focus on nursing education goals and incorporating them into curricula can be an effective step in educating people in the community. Thus, nursing professors and teachers can use the service-oriented learning model in practical and clinical courses offered for nursing students. Future studies can also address the effect of service-based learning on students’ knowledge, attitudes, performance, and educational competencies in different fields of medical sciences.

## Data Availability

All data generated or analyzed during this study will be available if necessary.
